# Biomimetic Upconversion Nanoparticles and Gold Nanoparticles for Novel Simultaneous Dual-Modal Imaging-Guided Photothermal Therapy of Cancer

**DOI:** 10.3390/cancers12113136

**Published:** 2020-10-27

**Authors:** Ruliang Wang, Han Yang, Rongxin Fu, Ya Su, Xue Lin, Xiangyu Jin, Wenli Du, Xiaohui Shan, Guoliang Huang

**Affiliations:** 1 Department of Biomedical Engineering, School of Medicine, Tsinghua University, Beijing 100084, China; wangrl13@mails.tsinghua.edu.cn (R.W.); yh18@mails.tsinghua.edu.cn (H.Y.); thu_frx@mail.tsinghua.edu.cn (R.F.); suya16@mails.tsinghua.edu.cn (Y.S.); lin-x15@mails.tsinghua.edu.cn (X.L.); jin-xy17@mails.tsinghua.edu.cn (X.J.); dwl19@mails.tsinghua.edu.cn (W.D.); sxh19@mails.tsinghua.edu.cn (X.S.); 2 National Engineering Research Center for Beijing Biochip Technology, Beijing 102206, China

**Keywords:** dual-modal imaging, gold nanoparticles, upconversion nanoparticles, cancer cell membrane, imaging-guided photothermal therapy

## Abstract

**Simple Summary:**

Multimodal imaging systems with high registration accuracy and molecular agents with highly specific targeting capacity are vital for imaging-guided theranostics of cancer. A novel simultaneous dual-modal imaging system combined with cancer cell membrane-coated nanoparticles as an imaging-guided photothermal therapy (PTT) was reported in this paper. A novel detector with the ability to detect both high-energy X-ray and low-energy visible light at the same time, as well as a dual-modal imaging system based on the detector, was developed for intrinsic simultaneous dual-modal imaging. Cancer cell membrane-coated upconversion nanoparticles (CC-UCNPs) and gold nanoparticles (CC-AuNPs) with the capacity for immune evasion and active tumor targeting were engineered for highly specific imaging and high-efficiency PTT therapy. The highly specific imaging-guided PTT efficacy was evaluated both in vitro and in vivo. All these results suggested our biomimetic UCNP/AuNP and novel simultaneous dual-modal imaging combination could be a promising platform and methodology for cancer theranostics.

**Abstract:**

Multimodal imaging-guided near-infrared (NIR) photothermal therapy (PTT) is an interesting and promising cancer theranostic method. However, most of the multimodal imaging systems provide structural and functional information used for imaging guidance separately by directly combining independent imaging systems with different detectors, and many problems arise when trying to fuse different modal images that are serially taken by inviting extra markers or image fusion algorithms. Further, most imaging and therapeutic agents passively target tumors through the enhanced permeability and retention (EPR) effect, which leads to low utilization efficiency. To address these problems and systematically improve the performance of the imaging-guided PTT methodology, we report a novel simultaneous dual-modal imaging system combined with cancer cell membrane-coated nanoparticles as a platform for PTT-based cancer theranostics. A novel detector with the ability to detect both high-energy X-ray and low-energy visible light at the same time, as well as a dual-modal imaging system based on the detector, was developed for simultaneous dual-modal imaging. Cancer cell membrane-coated upconversion nanoparticles (CC-UCNPs) and gold nanoparticles (CC-AuNPs) with the capacity for immune evasion and active tumor targeting were engineered for highly specific imaging and high-efficiency PTT therapy. In vitro and in vivo evaluation of macrophage escape and active homologous tumor targeting were performed. Cancer cell membrane-coated nanoparticles (CC-NPs) displayed excellent immune evasion ability, longer blood circulation time, and higher tumor targeting specificity compared to normal PEGylated nanoparticles, which led to highly specific upconversion luminescence (UCL) imaging and PTT-based anti-tumor efficacy. The anti-cancer efficacy of the dual-modal imaging-guided PTT was also evaluated both in vitro and in vivo. Dual-modal imaging yielded precise anatomical and functional information for the PTT process, and complete tumor ablation was achieved with CC-AuNPs. Our biomimetic UCNP/AuNP and novel simultaneous dual-modal imaging combination could be a promising platform and methodology for cancer theranostics.

## 1. Introduction

Cancer theranostics is widely recognized as a promising strategy to precisely fight cancer [1,2,3]. Among all theranostic methods, imaging-guided near-infrared (NIR) photothermal therapy (PTT) displays great advantages compared to traditional tumor ablation methods (e.g., chemotherapy and radiotherapy) and has attracted much attention due to its minimal invasiveness to normal tissues and deep tissue penetration capacity [4,5,6,7]. To improve the overall performance of imaging-guided PTT as a systematic method, research has mainly focused on three aspects: (1) photothermal reagents with higher photothermal conversion efficiency [3,8], (2) imaging reagents or methodology with higher tumor targeting specificity [6,9,10], and (3) multimodal imaging instruments that can provide both structural and functional information with higher sensitivity and spatial resolution to dynamically guide and monitor the entire therapy process [10,11,12].

Tumor-specific PTT is based on selective light absorption of abnormal cells at tumor sites that is induced by a specific targeting PTT reagent. Therefore, an ideal PTT reagent should be highly specific for tumor targeting and possess efficient photothermal converting activity. In the past few decades, a variety of PTT reagents, including organic polymers, carbon materials, and metal nanostructures, have been developed and used for tumor ablation [7,13,14,15,16,17]. Of all of these PTT reagents, gold nanoparticles (AuNPs) with tunable localized surface plasmon resonance (LSPR) peaks located at the NIR region have been actively studied [18,19,20]. Moreover, as an inert material, gold has been used in medicine for years due to its biocompatibility and non-toxicity.

Researchers have made efforts to enhance the PTT effect and overcome obstacles to make AuNPs more useful for clinical applications. To endow AuNPs with immune escaping ability and prolong their systemic circulation time so that they can accumulate at tumor sites through the enhanced permeability and retention effect (EPR) or active targeting mechanisms, biocompatible polymers (e.g., polyethylene glycol; PEG) are commonly used for the surface functionalization of AuNPs [21,22]. However, polymers still stimulate the immune system to different degrees, leading to clearing of the AuNPs from the body [23]. To specifically target cancer, a variety of surface modification strategies have been applied to nanoprobes. Most of them are ligand–receptor-mediated, relying on surface receptors that are overexpressed on cancer cells (e.g., folate (FA) with the FA receptor, anti-CD20 with CD20, or aptamers) [24,25,26,27,28]. However, these surface functionalization strategies are always chemically complicated and commonly lack high specificity due to the heterogeneity of tumor cells.

Different medical imaging modalities can be used to guide and assess the PTT process for cancer [2,4,5,29]. Among all the imaging modalities, multimodal imaging, which combines structural modality with functional modality, is a powerful and promising technique for biomedical and clinical applications [30,31,32]. For the functional imaging modality, nanoparticle-mediated fluorescent imaging methods have attracted much interest for their abundant biochemical and optical properties. Upconversion nanoparticles (UCNPs), particularly lanthanide-doped nanocrystals that convert NIR radiation to visible light, are a promising new generation of fluorescence nanoprobes for in vivo imaging. Compared to traditional Stokes-shifting fluorescent nanocrystals, UCNPs possess fascinating optical and biochemical features, such as deep light penetration depth in tissues, narrow emission spectrum peaks, low toxicity, and no autofluorescence [10,32,33,34,35]. However, to make full use of these properties, UCNPs face nearly the same problems of immune escape and active high-specificity tumor targeting as the AuNPs mentioned above.

With the rapid development of nanotechnology, biomimetic cancer cell membrane (CCM)-based nanoparticles have recently attracted considerable attention as imaging probes and therapeutic reagents [36,37,38,39,40,41]. CCM-camouflaged nanoparticles inherit the special properties of cancer cells, which are highly related to the cell membrane proteins, particularly immune escaping and homologous binding capabilities. Utilization of natural cell membranes for surface functionalization presents a novel top-down approach to yield complete replication of surface antigenic diversity from the source cells to the synthetic nanoparticles.

To fully improve the performance of a theranostic platform, multimodal imaging instrument systems play an important role. Many multimodal imaging systems have been reported and used for different theranostic applications. However, most of these reported multimodal imaging systems employ separated detectors for each modality, which makes the system relatively bulky and expensive [12]. Moreover, different detectors with different data formats and imaging geometries always require special algorithms or extra markers to calibrate different imaging spaces and merge multimodal information [12,42,43]. Thus, imaging performance such as resolution and registration accuracy always suffers some loss.

**Scheme 1:**
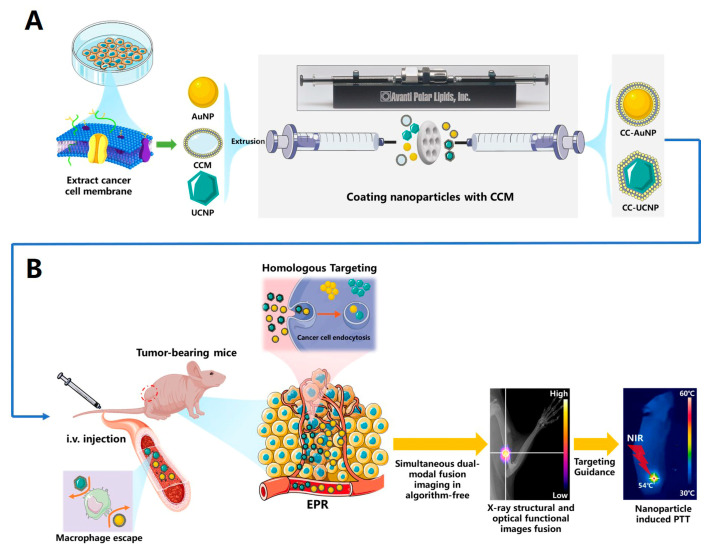
Schematic of the synthesis and application of cancer cell membrane-coated gold nanoparticles (CC-AuNPs) and cancer cell membrane-coated upconversion nanoparticles (CC-UCNPs) combined with a novel simultaneous dual-modal imaging platform for guided photothermal therapy (PTT) for cancer. The cancer cell membrane-coated UCNPs were used for highly specific tumor imaging and provided information for the PPT of cancer, which is based on the cancer cell membrane-coated AuNPs. (**A**) Preparation procedure of CC-NPs. (**B**) Schematic of immune escape and homologous targeting CC-NPs for dual-modal imaging-guided PTT.

Herein, to systematically improve the performance of the imaging-guided PTT methodology, we present a theranostic platform that combines CCM-based biomimetic UCNPs and AuNPs with a novel simultaneous dual-modal imaging system (Scheme 1). In this platform, AuNPs and UCNPs were cloaked with CCM (CC-AuNPs and CC-UCNPs) to enable immune escape and highly specific homologous cancer targeting (Scheme 1A). A dual-modal imaging system based on a novel detector (OptX) that is capable of detecting both high-energy X-rays and optical wavelength photons was designed and established. This system realized both upconversion fluorescent imaging and X-ray imaging with a single detector, and no extra marker or algorithm was needed for registration and merging of the two modalities. We demonstrate that CC-UCNPs could escape from the reticuloendothelial system (RES) and highly specifically target tumor sites. Together with X-ray imaging provided by the established imaging system, CC-UCNPs give information for the guidance and assessment of PTT therapy (Scheme 1B). We also demonstrate that CC-AuNPs as a PTT reagent performed much better than traditionally functionalized AuNPs. With all these integrated advantages, this study provides an outstanding methodology and platform for research on imaging-guided PTT for cancer.

## 2. Results

### 2.1. Synthesis and Characterization of CC-AuNPs and CC-UCNPs

Citrate-stabilized bare AuNPs and water-soluble β-NaYF_4_: Er^3+^, Yb^3+^ UCNPs with diameters of approximately 75 nm and concentrations of 50 μg·mL^−1^ and 2 mg·mL^−1^, respectively, were purchased from XFNANO. To fabricate CC-AuNPs and CC-UCNPs, the purified cancer cell membrane was first collected. Using HeLa cells as a model cancer cell line, membrane derivation and separation were achieved by emptying harvested cancer cells of their intracellular contents using a combination of hypotonic lysis, mechanical membrane disruption, and differential centrifugation. With the collected membrane, cancer cell membrane vesicles (CCV) were then formed by physical extrusion through a 400-nm porous polycarbonate membrane on a mini-extruder. Subsequently, the mixture of fresh CCV and prepared nanoparticles was repeatedly coextruded through a 200-nm porous polycarbonate membrane on a mini-extruder. Finally, the natural cancer cell membrane was functionalized onto AuNPs and UCNPs. The fabricated CC-AuNPs and CC-UCNPs were stored in PBS at 4 °C for further use.

**Figure 1:**
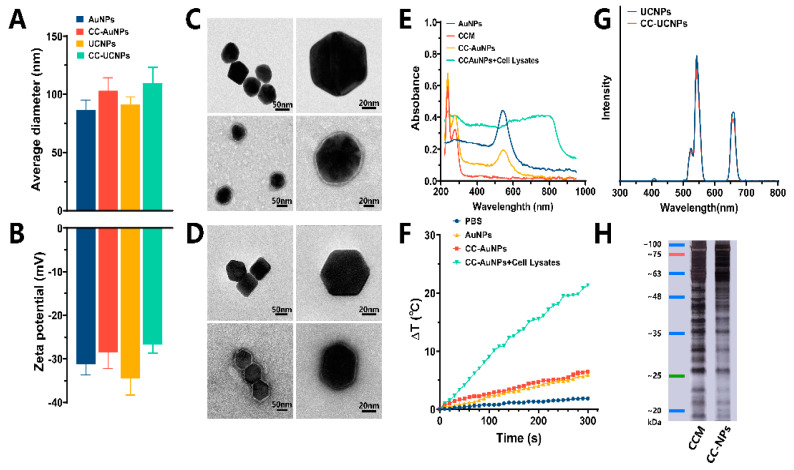
Characterization of CC-AuNPs and CC-UCNPs. (**A**) Hydrodynamic size and (**B**) zeta potential of AuNPs, CC-AuNPs, UCNPs, and CC-UCNPs. TEM images of (**C**) AuNPs, CC-AuNPs, (**D**) UCNPs, and CC-UCNPs. (**E**) UV–Vis–NIR spectra and (**F**) temperature change profile of AuNPs, CCM, CC-AuNPs, and CC-AuNPs with cell lysate. (**G**) Fluorescence spectra of UCNPs and CC-UCNPs. (**H**) SDS-PAGE protein analysis of cancer cell membrane (CCM) and CC-NPs. The data points represent the mean ± S.D. (*n* = 3).

Particle size (diameter, nm) and surface charge (zeta potential, mV) were measured by dynamic light scattering. The results indicated that the fabricated CC-AuNPs and CC-UCNPs were approximately 100 nm in diameter, and the zeta potential increased to approximately the level of CC-vesicles, suggesting a successful coating (Figure 1A,B). Furthermore, the morphologies of the CC-NPs were examined by transmission electron microscopy (TEM), and the TEM images display a nanoparticle core of approximately 75 nm in size and an outer lipid bilayer shell of approximately 10 nm in thickness (Figure 1C,D). UV–Vis–NIR spectra analysis showed that CC-AuNPs exhibit an LSPR peak at 546 nm, which is consistent with bare AuNPs. Additionally, two new absorption peaks at ~240 and 280 nm (which might match to the aromatic amino acids from membrane proteins) appeared in CCM and CC-AuNPs, further indicating the successful coating of CCM on AuNPs. The CC-AuNPs will aggregate after culturing with cells, which is induced by salt ions in the tumor interstitial fluid and cytoplasm, and the LSPR peak red-shifts to the NIR region [44]. HeLa cells were cultured with CC-AuNPs and then lysed, and the UV–Vis–NIR spectra of the lysate with CC-AuNPs displayed an obvious red-shift to the NIR region that is suitable for PTT (Figure 1E). The temperature change profile of PBS, AuNPs, CC-AuNPs, and CC-AuNPs cultured cell lysates solution in an EP tube under the irradiation of an NIR laser (808 nm, 2 W/cm^2^) also showed a significant higher PTT efficiency of CC-AuNPs cultured with cancer cells (Figure 1F). Meanwhile, fluorescence spectra analysis showed that CC-UCNPs exhibited fluorescence with typical emission peaks at 545 and 655 nm, and the cancer cell membrane coating had little influence on the fluorescence emission (Figure 1G).

It has been reported that the antigens on the cell membrane contribute to the immune escaping and tumor homologous targeting capacity of CC-NPs [45]. Protein gel electrophoresis was performed to validate the maintenance of membrane proteins after coating the AuNPs and UCNPs. SDS-PAGE analysis indicated that the protein profiles of CC-NPs coincided well with those of CCM, suggesting a good retention of the main membrane proteins after coating (Figure 1H).

### 2.2. Immune Evasion and Long-Term Circulation of CC-NPs

**Figure 2:**
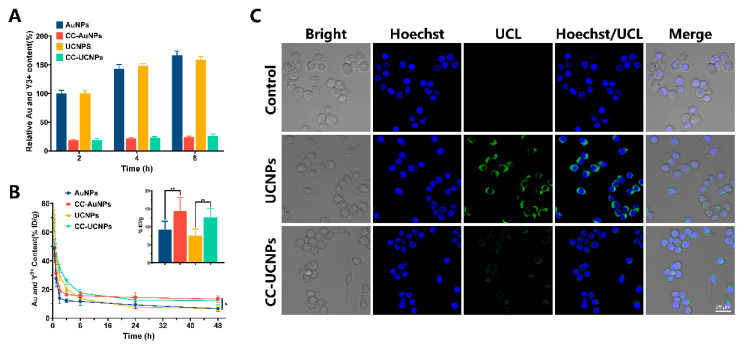
Immune evasion evaluation. (**A**) Quantitative analysis of the nanoparticle uptake of RAW264.7 cells with various incubation time periods. (**B**) In vivo pharmacokinetics curves over a span of 48 h after i.v. injection with various nanoparticles. (**C**) CLSM images of RAW 264.7 macrophage-like cells after incubation with various nanoparticles, scale bar = 25 μm. The data points represent the mean ± S.D. (*n* = 3), (*) indicates *p* < 0.05, (**) indicates *p* < 0.01.

To investigate the immune escaping capability of CC-NPs, RAW264.7 murine macrophage-like cells were used as a cell model. RAW264.7 cells were incubated with AuNPs, CC-AuNPs, UCNPs, and CC-UCNPs for various time intervals, and then intracellular Au content and Y3+ content was determined by inductively coupled mass spectroscopy (ICP-MS). The uptake of nanoparticles increased in a time-dependent manner. However, the intracellular nanoparticles in the CC-NPs-treated groups were significantly lower than those of the uncoated nanoparticles, suggesting the excellent immune evasion capacity of CCM-camouflaged nanoparticles (Figure 2A). Meanwhile, in vivo pharmacokinetics was performed in a BALB/c nude mouse model intravenously injected with AuNPs, CC-AuNPs, UCNPs, and CC-UCNPs. At various time points after injection, blood samples were collected for Au and Y3+ quantification by ICP-MS (Figure 2B). Compared to the uncoated nanoparticle-treated groups, the area under the curve (AUC_0~48_) of the CC-NPs-treated groups is significantly higher than that of NPs-treated groups, suggesting that the CCM coating prolonged the blood circulation and enhanced the blood retention of nanoparticles. Furthermore, RAW264.7 cells incubated with UCNPs and CC-UCNPs for 4h were also imaged with confocal laser scanning microscopy (CLSM) using a 980-nm NIR laser (Figure 2C). The cells treated with UCNPs exhibited bright green upconversion luminescence (UCL), while for CC-UCNPs, the UCL intensity was comparable to the control group, indicating that the CCM coating effectively reduced immune clearance.

### 2.3. Cancer Cell Homologous Targeting of CC-AuNPs and CC-UCNPs

**Figure 3:**
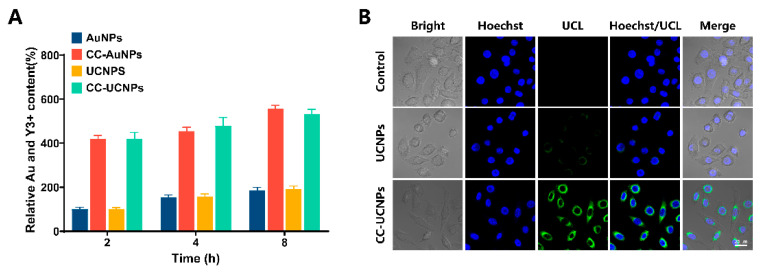
In vitro homologous targeting evaluation. (**A**) Quantitative analysis of the nanoparticle uptake of HeLa cells over various incubation time periods. (**B**) CLSM images of HeLa cells after incubation with various nanoparticles, scale bar = 25 μm. The data points represent the mean ± S.D. (*n* = 3).

It is reported that some cell surface proteins related to homologous cell adhesion contribute to the enhancement of the cellular uptake of CCM-coated nanoparticles [36,46,47]. To investigate the active cancer cell targeting capacity of CC-NPs, HeLa cells were incubated with AuNPs, CC-AuNPs, UCNPs, and CC-UCNPs for various time intervals, and the Au content and Y^3+^ content were determined by ICP-MS. Intracellular Au content and Y^3+^ content increased in a time-dependent manner in HeLa cells treated with AuNPs, CC-AuNPs, UCNPs, and CC-UCNPs. However, CC-AuNPs and CC-UCNPs exhibited higher levels of intracellular accumulation than AuNPs and UCNPs (Figure 3A). Subsequently, HeLa cells were incubated with UCNPs and CC-UCNPs for 4h and then imaged using CLSM under 980 nm NIR excitation. The cells treated with CC-UCNPs exhibited much brighter upconversion luminescence than the UCNPs-treated groups, which were comparable to the control group, indicating that the CCM coating effectively enhanced the homologous uptake of nanoparticles (Figure 3B).

### 2.4. In Vitro Photothermal Cytotoxicity of CC-AuNPs

**Figure 4:**
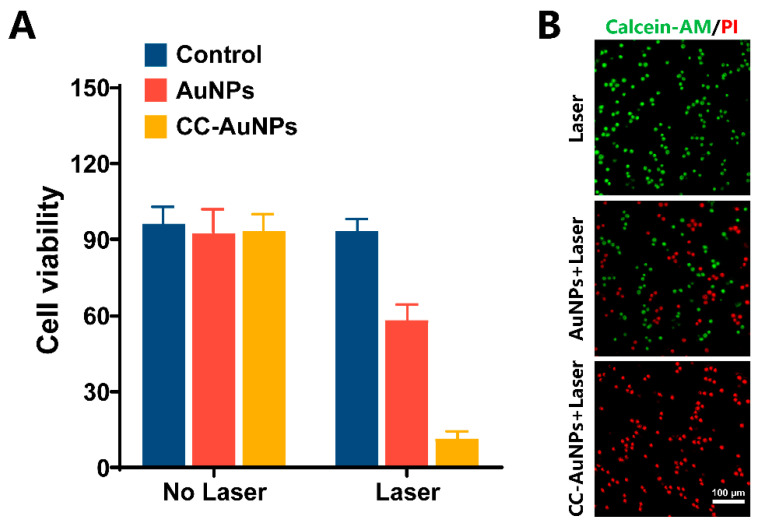
In vitro photothermal cytotoxicity evaluation. (**A**) Cell viability as measured by CCK-8 assay. (**B**) Fluorescent images of calcein-AM/PI-co-stained HeLa cells after different treatments, scale bar = 100 μm. The data points represent the mean ± S.D. (*n* = 3).

The in vitro photothermal cytotoxicity of CC-AuNPs to cancer cells was first evaluated using CCK-8 assays. HeLa cells treated with PBS, AuNPs, and CC-AuNPs without laser excitation (at an Au concentration of 50 μg/mL) caused ~5% cell death. This quite low toxicity to cells demonstrated the good biocompatibility of CCNPs. However, the viability of the same cell groups treated with the NIR laser (808 nm, 3 W/cm^2^, 5 min) decreased to 58 and 12%, respectively (Figure 4A). These results suggest that the photothermal efficacy was greatly enhanced with the increased intracellular Au concentration due to the homologous targeting of CC-AuNPs. Simultaneous fluorescence staining of live/dead cells using Calcein-AM and propidium iodide (PI) further proved the excellent photothermal efficacy of CC-AuNPs with the NIR laser (Figure 4B).

### 2.5. In Vivo Dual-Modal Imaging of Highly Specific Tumor Targeting

**Figure 5:**
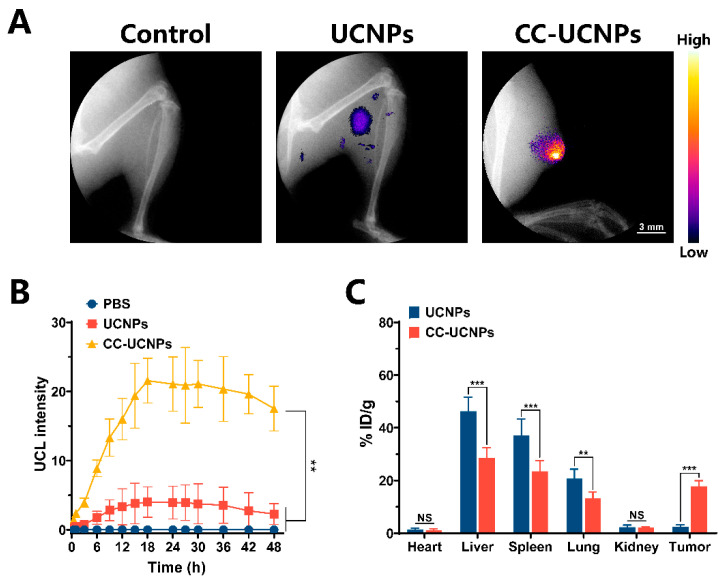
In vivo highly specific dual-modal imaging evaluation. (**A**) In vivo dual-modal imaging of BALB/c nude mice bearing HeLa cervical tumor xenografts at 18 h after i.v. injection of PBS or PBS containing UCNPs and CC-UCNPs. (**B**) An upconversion luminescence (UCL) intensity curve at the tumor site over a time span of 48 h. (**C**) Biodistribution of UCNPs and CC-UCNPs at 18 h after the i.v. injection. The data points represent the mean ± S.D. (*n* = 4). (**) indicates *p* < 0.01, and (***) indicates *p* < 0.001.

To further evaluate the tumor targeting capacity-based highly specific UCL imaging of CC-UCNPs in vivo, BALB/c nude mice bearing HeLa cervical tumor xenografts (*n* = 4) received an intravenous (i.v.) injection of PBS or PBS containing UCNPs and CC-UCNPs. By using the self-constructed novel X-ray/UCL dual-modal imaging system, high-resolution and low-background dual-modal imaging of the tumor site was performed at different time points in all group sets (Figure 5A). At each time point, the group treated with CC-UCNPs exhibited a much higher UCL signal than groups treated with PBS and UCNPs. After the injection, the UCL signal of the CC-UCNPs-treated group first increased in a time-dependent manner, reaching a plateau at ~18 h, then slowly decreased after 30 h, which indicated that the tumor targeting and aggregation process of CC-NPs reached the highest level at the time point of approximately 18 h after injection (Figure 5B). This gives time point guidance for the beginning of the following PTT therapy. Subsequently, all of the mice were sacrificed, and the tumors and major organs were collected to quantitatively analyze the biodistribution of different types of nanoparticles using ICP-MS. Compared to the UCNPs-treated groups, the CC-UCNPs-treated mice exhibited significantly higher accumulation in the tumor and lower accumulation in the spleen and liver, indicating the homologous in vivo targeting capacity of CCM-coated nanoparticles and the highly specific tumor imaging property of CC-UCNPs (Figure 5C).

### 2.6. In Vivo Anti-Tumor PPT

**Figure 6:**
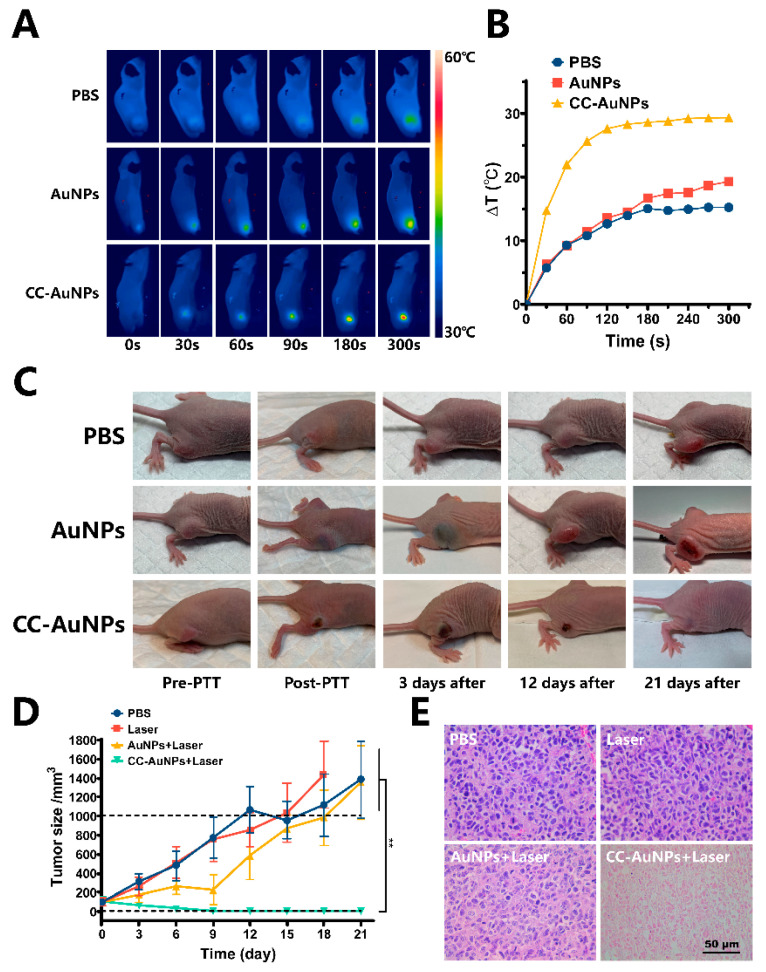
In vivo anti-tumor PTT efficacy. (**A**) IR thermal images of BALB/c nude mice bearing HeLa cervical tumor xenografts. (**B**) Temperature change profile at tumor site of different groups. (**C**) Representative photos of BALB/c nude mice bearing HeLa cervical tumor xenografts over a time span of 21 days. (**D**) Tumor growth curves of different groups after treatments. (**E**) H&E staining of the tumors after the PTT procedure. Scale bar = 50 μm. The data points represent the mean ± S.D. (*n* = 4). (**) indicates *p* < 0.01.

To validate the enhanced accumulation and aggregation of CC-AuNPs, the in vivo PTT effect was evaluated in tumor-bearing BALB/c nude mice. BALB/c nude mice bearing HeLa cervical tumor xenografts (*n* = 4) received an intravenous injection of PBS or PBS containing AuNPs and CC-AuNPs. Guided by the highly specific dual-modal imaging, PTT was conducted 18 h after the injection, and the temperature of the tumor sites was evaluated by an infrared (IR) camera and the temperature change profile at tumor site was recorded during the NIR laser treatment (Figure 6A,B). The temperature at the tumor site of the CC-AuNPs-treated group rapidly reached 55.5 °C, which is high enough to kill cancer cells. In contrast, in the PBS- or PBS containing AuNPs-treated groups, the temperature only reached 41.7 and 45.8 °C, respectively, which is not high enough for tumor therapy. To further investigate the PTT effect in vivo, long-term tumor growth curves were measured. In the CC-AuNPs-treated group, after the PTT therapy process, the tumors were effectively ablated with no recurrence and only a scar was left at the original tumor site (Figure 6C). In contrast, in the PBS containing AuNPs-treated groups, the tumor growth was initially inhibited, but recurrence was observed over the following days. As expected, in the PBS-treated group, no inhibition of tumor growth was observed, and the tumor size reached >1000 mm^3^ 21 days after the administration (Figure 6D). Hematoxylin and eosin (H&E) analysis was also used to validate the cell damage effect after PTT treatment. We found that there was significant cell damage in the CC-AuNPs-treated group, while only moderate or nearly no damage was observed in the PBS- and AuNPs-treated groups (Figure 6E). Taken together, these results indicate that cancer cell-camouflaged AuNPs were more effective for PTT than AuNPs with traditional surface modification.

### 2.7. In Vivo Toxicity Evaluation

**Figure 7:**
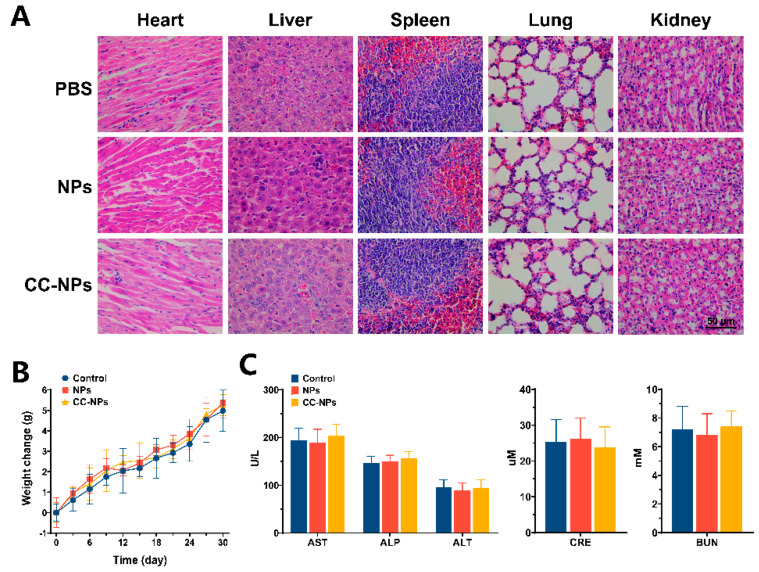
In vivo toxicity evaluation. (**A**) H&E staining of major organs. (**B**) Body weight change curves of mice treated with different nanoparticles over a span of 30 days. Scale bar = 50 μm. (**C**) Blood chemistry data. Liver function markers: AST, ALP, and ALT; kidney function markers: BUN and CRE. The data points represent the mean ± S.D. (*n* = 5).

The in vivo toxicity of nanomaterials is always a concern for biological application, especially for unbiodegradable materials. Herein, BALB/c nude mice (*n* = 5) received i.v. injection of 200 µL of PBS, PBS containing NPs, or CC-NPs (serial injection of two kinds of nanoparticles) at a concentration of 5 mg mL^−1^ for toxicity evaluation. Body weight change is always an indicator for in vivo toxicity. No significant difference in body weight change was observed between the different groups over a span of 30 days (Figure 7B), indicating that these nanoparticles have no obvious toxicity to mice. Thirty days post-injection, all mice were sacrificed, and their blood and major organs were collected for blood biochemistry, hematology tests, and histology analysis. No noticeable signs of significant major organ damage were observed in H&E-stained tissue slices (Figure 7A). Furthermore, the blood parameters and blood biochemistry indicators (i.e., blood urea nitrogen (BUN), creatinine (CRE), alanine transaminase (ALT), aspartate aminotransferase (AST), and alkaline phosphatase (ALP)) showed no significant differences between the treatment groups and the control group (Figure 7C). Although more systematic studies of short- and long-term toxicity are needed, all of the results of our small-scale pilot study preliminary indicate low in vivo toxicity and good biocompatibility of the CC-NPs.

## 3. Materials and Methods

### 3.1. Materials

Phosphate buffer saline (PBS) was purchased from Hyclone Laboratories Inc. (Chicago, IL, USA). Ethylenediaminetetraacetic-acid (EDTA), Cell Counting Kit-8 (CCK-8), and uranyl acetate were obtained from Sigma-Aldrich (St. Louis, MO, USA). EDTA-free mini protease inhibitor tablets were obtained from Roche (Basel, Switzerland). AuNPs, UCNPs, PEG-AuNPs, and PEG-UCNPs were purchased from XFNano (Nanjing, China). The RPMI 1640 medium and Hoechst 33,342 were purchased from Yeasen (Shanghai, China). All of the aqueous solutions were prepared using deionized water (DI water) produced with a Milli-Q purification system.

### 3.2. Cell and Animal Models

HeLa cells and RAW 264.7 murine macrophage-like cells were provided by the cell bank of the Chinese Academy of Medical Sciences. The cells were cultured in the standard cell medium recommended by the American Type Culture Collection (ATCC) at 37 °C in a 5% CO_2_ atmosphere.

BALB/c nude mice were housed in isolated ventilated cages (maximum of six mice per cage) in the barrier facility at Tsinghua University. The mice were maintained on a 12/12-h light/dark cycle at 22–26 °C with sterile pellet food and water ad libitum. BALB/c nude mice bearing HeLa cervical tumor xenografts were obtained by subcutaneous (s.c.) injection of 50 μL serum-free cell medium containing 5 × 10^6^ HeLa cells into the leg of each mice. After the tumor volume reached ~50 mm^3^, the tumor-bearing mice were used for further experiments.

The laboratory animal facility has been accredited by the AAALAC (Association for Assessment and Accreditation of Laboratory Animal Care International), and the IACUC (Institutional Animal Care and Use Committee) of Tsinghua University approved all animal protocols used in this study (ethic approval number: 16-HGL1).

### 3.3. Self-Constructed Novel Simultaneous Dual-Modal In Vivo Imaging System

**Figure 8:**
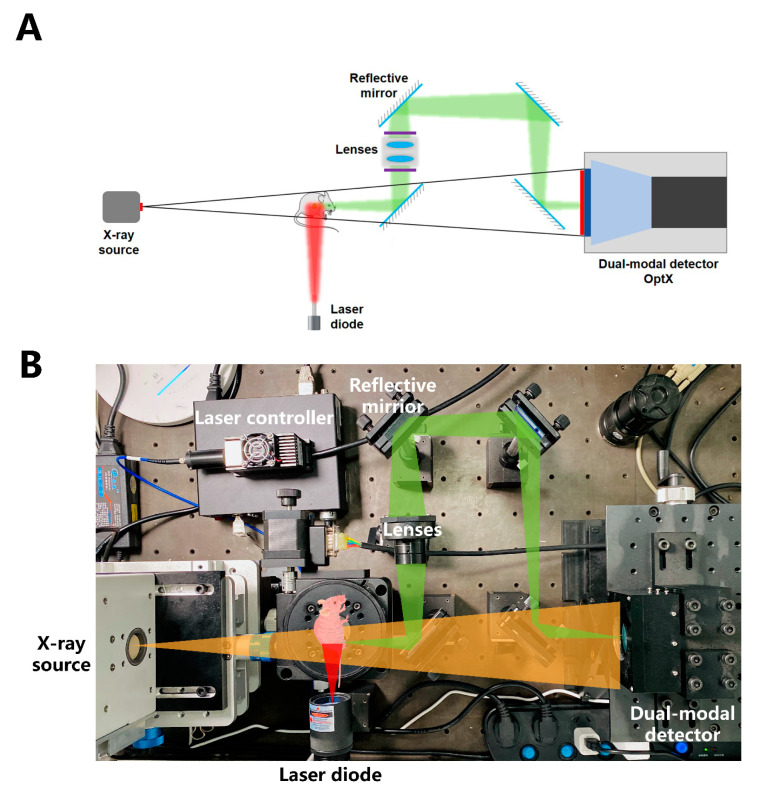
Schematic illustration and photo of the novel simultaneous dual-modal in vivo imaging system setup. (**A**) The design structure of the simultaneous dual-modal in vivo imaging system. (**B**) Photo of the simultaneous dual-modal in vivo imaging system.

The novel simultaneous dual-modal in vivo imaging system used in this paper is a self-constructed imaging platform. The scheme and photo of the system setup is as shown below (Figure 8). Most of the reported dual-modal in vivo imaging systems are a combination of individual detectors set around the imaging target. Due to this mainstream type of setup, there is an inherent angle bias of the two imaging modalities, and extra marks or a registration algorithm are needed to fuse the multimodal images produced by the different detectors with different relative positions. In this study, with a self-constructed novel detector that can detect both high-energy X-rays and low-energy visible light at the same time, a dual-modal imaging system with the ability to simultaneously take a digital radiograph and a fluorescent image was established. A microfocal spot X-ray source (Trinity 9100004, Oxford Instrument Inc., Abingdon, United Kingdom) irradiates a 33-μm cone beam through the animal to generate a high-resolution digital radiograph. At the same time, a 980-nm laser diode excites the UCNPs on the tumor site, and the upconversion fluorescent signal is collected by the detector through a paraxial light path with a thin reflective mirror and objective lenses. Two modal images are produced from one single detector with almost no position and time bias, which means no extra markers, prior information, or complicated registration algorithm are needed to accurately fuse different modal images with high resolution.

**Figure 9:**
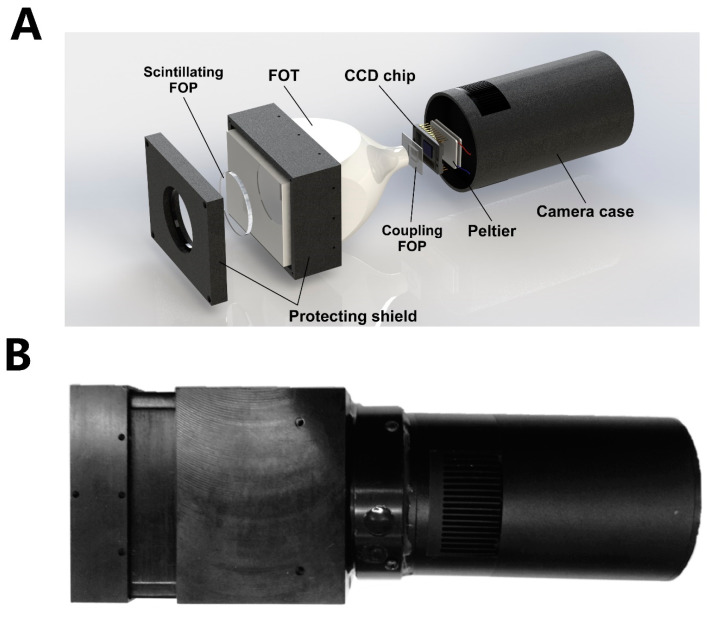
Illustration of the design structure and photo of the self-constructed dual-modal OptX detector. (**A**) The design structure of the dual-modal OptX detector. (**B**) Photo of the dual-modal OptX detector.

The key component of this novel imaging system is the self-constructed dual-modal OptX detector, the design structure and photo of which are shown below (Figure 9). The detector utilizes a commercial CCD chip (ICX285AL, SONY) as the image sensor and Peltier chilling plate-based refrigeration circuits to build a low-noise and high-resolution B/W cooling camera. As the size of the sensing area (10.2 × 8.3 mm) is too small for radiograph imaging, an ultra-high-magnification ratio (up to 6.97:1) fiber optic taper (FOT) was coupled to the silicon surface of the sensor in Figure 9A. The quartz glass shield of the CCD sensor was removed, and a customized fiber optic plate (FOP) was bonded between the FOT and the sensing area by UV-curing gel. To endow the camera with the ability to detect both high-energy X-rays and low-energy visible light, a scintillating FOP made with scintillating glass (Tb3+-doped silicate glass) was coupled to the large end of the FOT to covert high-energy X-rays to visible light that could be transmitted through the FOT and be detected by the CCD camera. For the fluorescence detection, the scintillating FOP serves as a transparent image transfer medium to transmit the optical image plane to the CCD sensing plane.

**Figure 10:**
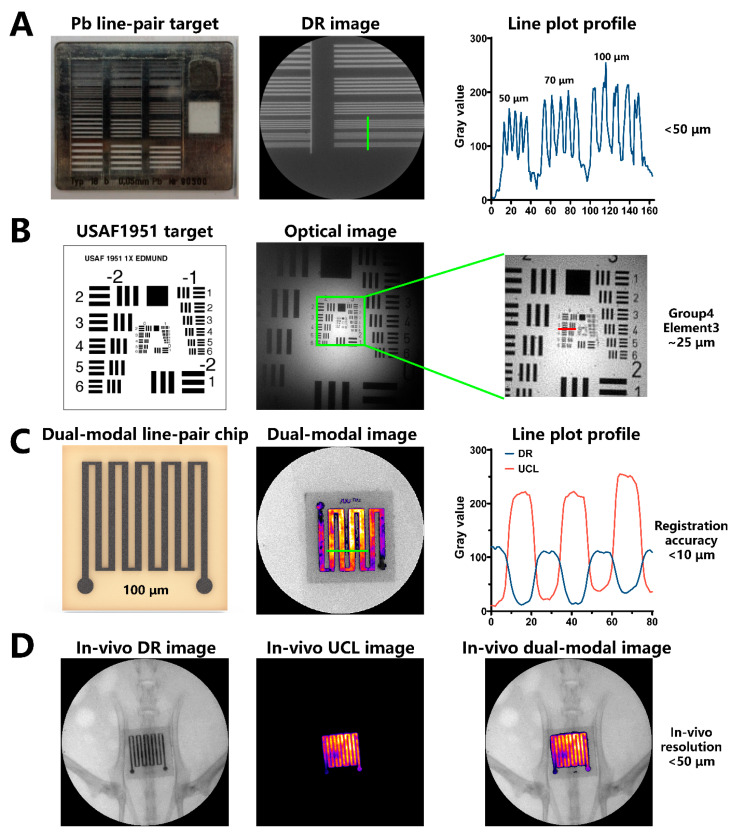
Performance evaluation of the dual-modal imaging system. (**A**) Resolution test of the digital radiograph modality using a Type-18b Pb line-pair target; the digital radiograph image resolution was <50 μm. (**B**) Resolution test of the optical imaging modality using a USAF 1951 testing target; the optical resolution was 25 μm. (**C**) Dual-modal registration accuracy test using a homemade dual-modal line-pair chip; the registration accuracy was <10 μm. (**D**) In vivo dual-modal imaging performance tested by subcutaneously implanting the dual-modal line-pair chip in a mouse; the in vivo resolution was 50 μm.

With this novel dual-modal detector and an imaging system based on it, the UCL imaging and X-ray radiograph of the small animals in this study were simultaneously taken and directly fused by pixel-to-pixel overlap of dual-modal images from one detector for the image properties (resolution, bit depth, and spatial position) of the two modal images to be totally the same. The resolution of two separate imaging modalities was evaluated using a Type-18b Pb line-pair and USAF 1951 testing target, and we found that the radiographic and optical resolution could reach >50 and 25 μm (Figure 10A,B). There is no commercial standard imaging target for the evaluation of the registration accuracy of the dual-modal image fusion, so a microfluidics line-pair chip fabricated using lithography technology was used as an evaluation target. The microchannel of the chip, with a width ranging 25~500 μm, was filled with a mixture of UCNPs and Pb powder for dual-modal imaging. Dual-modal images of this homemade evaluation target (dual-modal line-pair chip) were captured with the novel imaging platform. Theoretically, when the registration is perfect, the radiographic signal and the UCNPs signal of the channel match exactly with no pixel bias. The gray value line plots of separated dual-modal images at the line-pair site were tested, and we found that the registration accuracy of the self-constructed imaging platform was <10 μm, which is much higher than other reported systems (Figure 10C). Meanwhile, the chip was subcutaneously implanted in a mouse body, and the in vivo dual-modal imaging performance was tested. With the help of a tissue optical clearing reagent, the in vivo resolution was 50 μm (Figure 10D).

### 3.4. Statistical Analysis

All data are shown as the mean ± SD, and all analyses were performed using GraphPad Prism 8.0. Two-tailed, unpaired Student’s *t*-tests were used for statistical analysis. *p* < 0.05 indicates statistical significance.

## 4. Discussion

Tumor-specific PTT is a promising theranostic method to fight cancer. Both highly specific tumor targeting reagents and imaging system of high performance are critical to the improving of the overall performance of the imaging-guided PTT methodology. The results above have shown that a highly specific imaging-guided high-efficiency PTT therapy of cancer was achieved by combining CCM-biomimetic nanotechnology and simultaneous dual-modal imaging system together.

The reported novel simultaneous dual-modal imaging system provided a more convenient and precise imaging system prototype for small animal research. With the self-constructed dual-modal detector, no extra complicated registration algorithm is needed for the fusion of a digital radiograph and a fluorescent imaging modality with higher registration accuracy. Both in vitro cell line and in vivo small animal research proved that CCM-coating nanoparticles inherited the capability of immune escaping and homologous tumor targeting from cancer cell. The improved blood retention time and targeting efficiency eventually leads to a more specific imaging performance and PTT efficiency which also were proved by small animal model research.

These results suggested that the proposed biomimetic UCNP/AuNP and novel simultaneous dual-modal imaging combination is very promising for cancer research. However, more issues should be investigated further and more improvements could be achieved. For example, the field of view (FOV) of the current imaging system is relatively small for a whole-body imaging, further research on developing new detector with bigger FOV is very valuable. Cell membrane from different types of cell source may endow nanoparticles with different properties, further research on developing biomimetic nanomaterials with more useful biomedical capability deserve expectation. For the concern of biosafety, long-term toxicity evaluation of these nanomaterials is very necessary.

## 5. Conclusions

In summary, we report a novel dual-modal imaging system based on a self-developed detector that can simultaneously record a digital radiograph and a fluorescent image without any extra registration algorithm for the fusion of these two imaging modalities. The performance test results suggested a high resolution of each modality and a high registration accuracy of the imaging system. By introducing a CCM-biomimetic nanotechnology, CCM-coated UCNPs and AuNPs were developed for the imaging-guided PTT of cancer. Combining the novel imaging system with the CCM-based biomimetic nanotechnology, an excellent complete theranostic methodology for cancer was demonstrated. The CCM-coated nanoparticles exhibited excellent immune escaping and homologous targeting compacities. The highly specific and sensitive functional information provided by CC-UCNPs and high-resolution structural information provided by the digital radiograph yielded a precise time point and position guidance for the PTT of cancer. The high accumulation and aggregation of CC-AuNPs at tumor sites provided an excellent photothermal efficiency for the thermal ablation of cancer. Overall, we aimed to fully improve the performance of PTT-based theranostic systems by introducing a novel dual-modal imaging system combined with the CCM-based biomimetic nanotechnology. Although it is only a proof-of-concept attempt of this methodology and more issues need to be systematically investigated, we have reason to believe that the novel imaging system combining with the cell membrane-based biomimetic nanotechnology could open up a new area in precise diagnosis and therapy for cancer.
